# A flagellar accessory protein links chemotaxis to surface sensing

**DOI:** 10.1128/jb.00404-24

**Published:** 2024-10-18

**Authors:** Rachel I. Salemi, Ana K. Cruz, David M. Hershey

**Affiliations:** 1 Department of Bacteriology, University of Wisconsin-Madison, Madison, Wisconsin, USA; Queen Mary University of London, London, United Kingdom

**Keywords:** surface sensing, *Caulobacter*, flagellum, signaling network, chemotaxis, c-di-GMP, bacterial behavior, surface colonization

## Abstract

Bacteria find suitable locations for colonization by sensing and responding to surfaces. Complex signaling repertoires control surface colonization, and surface contact sensing by the flagellum plays a central role in activating colonization programs. *Caulobacter crescentus* adheres to surfaces using a polysaccharide adhesin called the holdfast. In *C. crescentus*, disruption of the flagellum through interactions with a surface or mutation of flagellar genes increases holdfast production. Our group previously identified several *C. crescentus* genes involved in flagellar surface sensing. One of these, *fssF*, codes for a protein with homology to the flagellar C-ring protein FliN. We show here that a fluorescently tagged FssF protein localizes to the flagellated pole of the cell and requires all components of the flagellar C-ring for proper localization, supporting the model that FssF associates with the C-ring. Deleting *fssF* results in a severe motility defect, which we show is due to a disruption of chemotaxis. Epistasis experiments demonstrate that *fssF* promotes adhesion through a stator-dependent pathway when late-stage flagellar mutants are disrupted. Separately, we find that disruption of chemotaxis through deletion of *fssF* or other chemotaxis genes results in a hyperadhesion phenotype. Key genes in the surface sensing network (*pleD*, *motB*, and *dgcB*) contribute to both ∆*flgH-*dependent and ∆*fssF-*dependent hyperadhesion, but these genes affect adhesion differently in the two hyperadhesive backgrounds. Our results support a model in which the stator subunits of the flagella incorporate both mechanical and chemical signals to regulate adhesion.

**IMPORTANCE:**

Bacterial biofilms pose a threat in clinical and industrial settings. Surface sensing is one of the first steps in biofilm formation. Studying surface sensing can improve our understanding of biofilm formation and develop preventative strategies. In this study, we use the freshwater bacterium *Caulobacter crescentus* to study surface sensing and the regulation of surface attachment. We characterize a previously unstudied gene, *fssF*, and find that it localizes to the cell pole in the presence of three proteins that make up a component of the flagellum called the C-ring. Additionally, we find that *fssF* is required for chemotaxis behavior but dispensable for swimming motility. Lastly, our results indicate that deletion of *fssF* and other genes required for chemotaxis results in a hyperadhesive phenotype. These results support that surface sensing requires chemotaxis for a robust response to a surface.

## INTRODUCTION

Bacteria frequently encounter solid surfaces in their surroundings that offer favorable habitats for colonization (1–3). Attachment to surfaces provides improved access to nutrient resources and protection from environmental stressors. These benefits are vital for growth and fitness in many bacteria. As bacteria undergo development, they also transition from a motile state to a sessile, attached state (4). The lifestyle involving attachment to surfaces poses a significant threat in clinical and industrial settings (5). Understanding the mechanisms underlying surface colonization can inform new strategies to control bacterial colonization.

**Fig 1:**
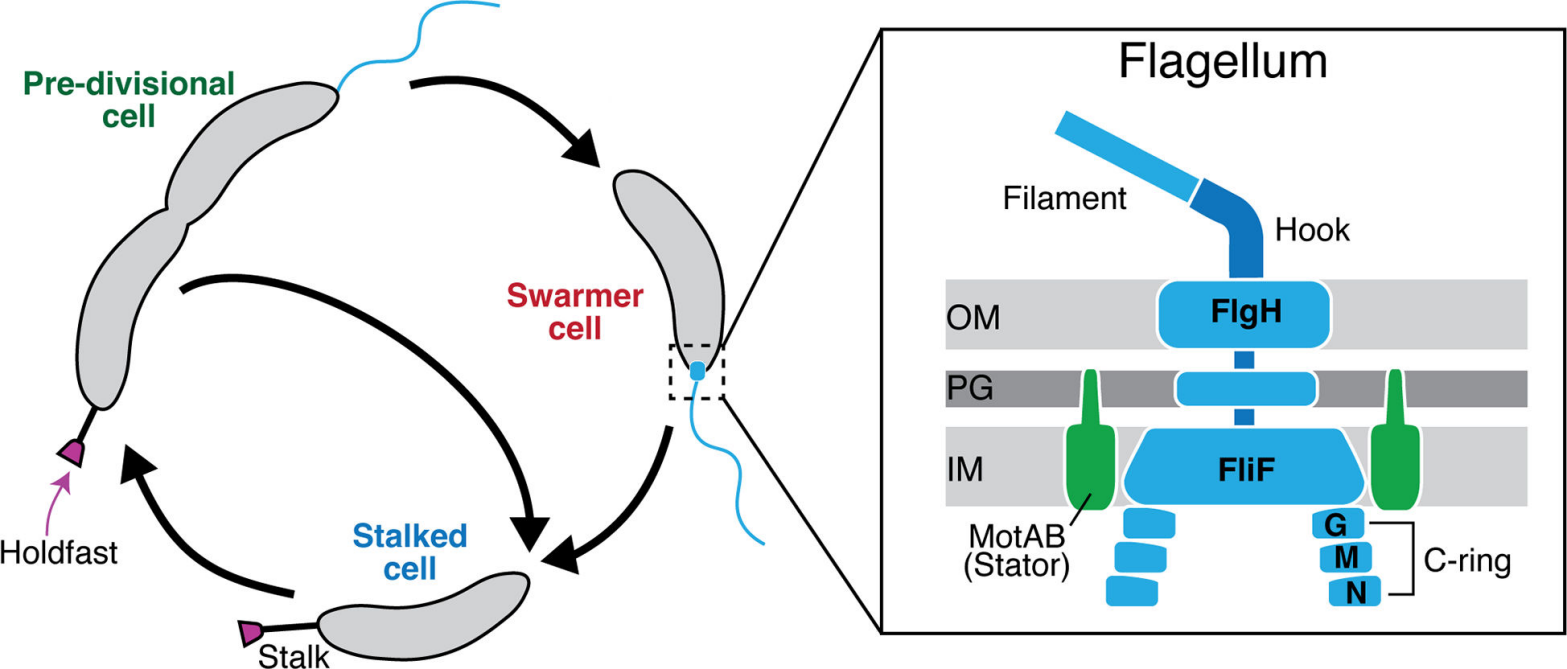
The *Caulobacter crescentus* flagellum is a complex multimeric machine. *C. crescentus* cells begin as a motile swarmer cell displaying a flagellum but are unable to reproduce. Differentiation into a stalked cell allows the cell cycle to continue and replication to begin. During this differentiation, the flagellum is lost and replaced by a stalk. Stalked cells can also produce an adhesive polysaccharide called the holdfast, which allows them to irreversibly attach to a surface. Stalked cells divide to release a swarmer cell into the environment. Flagella are assembled at the incipient swarmer pole in predivisional cells and disassembled as swarmer cells transition to become stalked cells. The flagellum is composed of many subunits and spans the entire cell envelope. The cytoplasmic C-Ring is composed of three proteins that bind in a hierarchical manner, FliG (**G**), FliM (**M**), and FliN (**N**). The MS-ring spans the inner membrane and comprises the protein FliF. The L-Ring spanning the outer membrane comprises the protein FlgH.

The motile-to-sessile transition requires signaling pathways that respond to physical contact with surfaces. Many bacteria use a molecular machine called the flagellum to recognize surface contact (6–8) (Fig. 1). This sophisticated trans-envelope complex is best known for its role in cellular motility. The flagellar components are typically divided into classes based on an assembly hierarchy. Each class of flagellar genes requires proper expression and assembly of the previous class in the hierarchy (6, 9–11) (Fig. 1). The flagellar motor contains a cytoplasmic-ring (C-ring) composed of FliG, FliM, and FliN and a membrane-spanning ring (MS-ring) composed of FliF that form the rotor complex (6, 10, 12–17) (Fig. 1). Ion channels called stators engage with the C-ring to turn the rotor and its associated filament (12, 18). Stator subcomplexes MotA and MotB rotate the motor (12, 14, 19, 20).

The direction of motor rotation is regulated by a behavior called chemotaxis. Chemotaxis allows cells to move toward attractants or away from repellents (12). The *Escherichia coli* chemotaxis system utilizes receptors called methyl-accepting chemotaxis proteins (MCPs) that detect attractants in the environment (21–24). Attractant binding to MCPs controls a histidine kinase, CheA, that phosphorylates the response regulator protein CheY (14, 22, 25). CheY-P translocates to the flagellar motor and interacts with the C-Ring proteins FliM and FliN (8, 14, 26). When CheY-P binds, the C-Ring expands, altering the interaction with MotAB from the interior of the ring to the exterior of the ring. This interaction disrupts the smooth swimming pattern by switching the direction of the motor, causing the cells to tumble and reorient (12, 14, 19, 20). While how chemotaxis signaling affects motility is well-established, less is known about how chemotaxis regulates other processes such as surface colonization.

The rotation of the flagellum becomes physically obstructed during surface encounters (27). This blocked state activates signaling pathways that promote surface adaptation by altering the motility behavior, increasing irreversible attachment, and stimulating biofilm formation in a variety of bacterial species (6, 7, 28). The intracellular second messenger cyclic di-guanosine monophosphate (c-di-GMP) plays a central role in promoting surface adaptation (Romling *et al*. 2013; Jenal *et al*. 2017; Valentini and Filloux 2016). High concentrations of c-di-GMP increase biofilm formation and sessile behaviors, while low concentrations are associated with increased motility. Cellular concentrations of c-di-GMP are regulated by two enzyme families: iguanylate cyclases (DGCs) and phosphodiesterases (PDEs), which, respectively, synthesize and degrade c-di-GMP (29). Though the enzymatic activity of DGCs is well-studied, the flagellum regulates c-di-GMP production through complex signaling pathways that have been difficult to disentangle (30).

*Caulobacter crescentus* is a robust model organism for studying surface colonization (7, 31). This Gram-negative, alphaproteobacterium is abundant in freshwater systems (31). During the *C. crescentus* cell cycle, sessile cells called stalked cells divide asymmetrically to release nonreplicative swarmer cells (Fig. 1) (32). Swarmer cells harbor a flagellum and pili at the old cell pole, which are used for motility and surface sensing (33–35). Swarmer cells must differentiate into stalked cells before they can replicate (36). Swarmer cells transition to become stalked cells by releasing their flagella, retracting their pili, synthesizing a stalk, and initiating chromosome replication (35). During the swarmer-to-stalked cell transition, *C. crescentus* cells can synthesize a specialized adhesin called the holdfast at the stalked cell pole (7, 37, 38). Surface sensing stimulates adhesion by increasing the likelihood that stalked cells will assemble a holdfast, making holdfast production an ideal phenotype for dissecting surface sensing (28, 38).

We previously identified a large set of *C. crescentus* genes involved in signaling pathways activated by the flagellum (28). Mutations in flagellar assembly genes stimulate the surface sensing pathway, indicating that disrupting the flagellar motor through mutation can mimic mechanical cues for surface contact. The resulting gain-of-function phenotype was used to identify *flagellar signaling suppressor* (*fss*) mutations that restore wild-type levels of adhesion in a hyper-adhesive flagellar mutant background (∆*flgH*). These novel surface sensing genes make up two separate pathways. A “developmental” pathway requiring the DGC *pleD* is activated when the flagellar assembly is disrupted at any stage. A second, “mechanical” pathway requiring the MotAB stators and the stator-associated DGC *dgcB* is activated specifically in late-stage flagellar mutants that retain an intact motor complex. Deletion of genes required for early stages of flagellar assembly (i.e., *fliF*, *fliG*, *fliN*, and *fliM*) results in a hyperadhesivie phenotype that is entirely dependent on the developmental pathway. Deletion of genes required for assembly of the hook and filament (i.e., *flgH*) results in a hyperadhesive phenotype that involves both the developmental and mechanical pathways (refer to Fig. 4A for the regulatory schematic).

In this study, we have examined a surface sensing factor called *fssF* (CC_2175, CCNA_02257) that contributes to holdfast stimulation downstream of the ∆*flgH* mutation. FssF shows high sequence homology to the C-Ring protein FliN, and we found that FssF localizes to the pole of *C. crescentus* cells in a C-Ring-dependent manner. Mutating *fssF* abolishes the ability of cells to migrate through soft agar and eliminates chemotaxis behavior in liquid. When adhesion is activated by deletion of *flgH,* we find that *fssF* contributes to the stator-dependent, mechanical pathway. Separately, we find that ∆*fssF* and other mutants that cannot perform chemotaxis exhibit a hyperadhesive phenotype that is distinct from that of flagellar mutants. Hyperadhesion in Che– mutants is entirely dependent on *motB* for activation. This work expands on the understanding of bacterial surface responses by indicating that the flagellar motor integrates mechanical and chemical cues to promote surface colonization.

## RESULTS

### Uncharacterized FliN homolog *fssF* suppresses ∆*flgH-*induced hyperadhesion

Deleting *flgH* or other flagellar genes causes a hyperadhesive phenotype in *C. crescentus* (39). To identify genes required for this hyperadhesion phenotype, we previously performed adhesion profiling of a ∆*flgH* transposon library (28). One of the genes identified through this screening was an uncharacterized gene that we named *fssF* (*flagellar signaling suppressor F*) (Fig. S1A). *fssF* codes for a predicted 103-amino acid protein. To further understand the function of FssF, we compared the protein sequences of other annotated flagellar proteins and found that the protein shows homology to FliN proteins from many bacteria, suggesting that *C. crescentus* contains two FliN homologs (Fig. S1B). Additionally, we found that this gene was conserved among most members of the *Caulobacter*. While the core C-Ring proteins are conserved among flagellated bacteria, some C-Ring complexes contain accessory proteins that perform specialized roles (13, 40). We hypothesized due to its similarity with FliN that FssF may localize to the C-Ring and contribute to motility.

### C-Ring proteins are required for FssF-Venus localization

**Fig 2:**
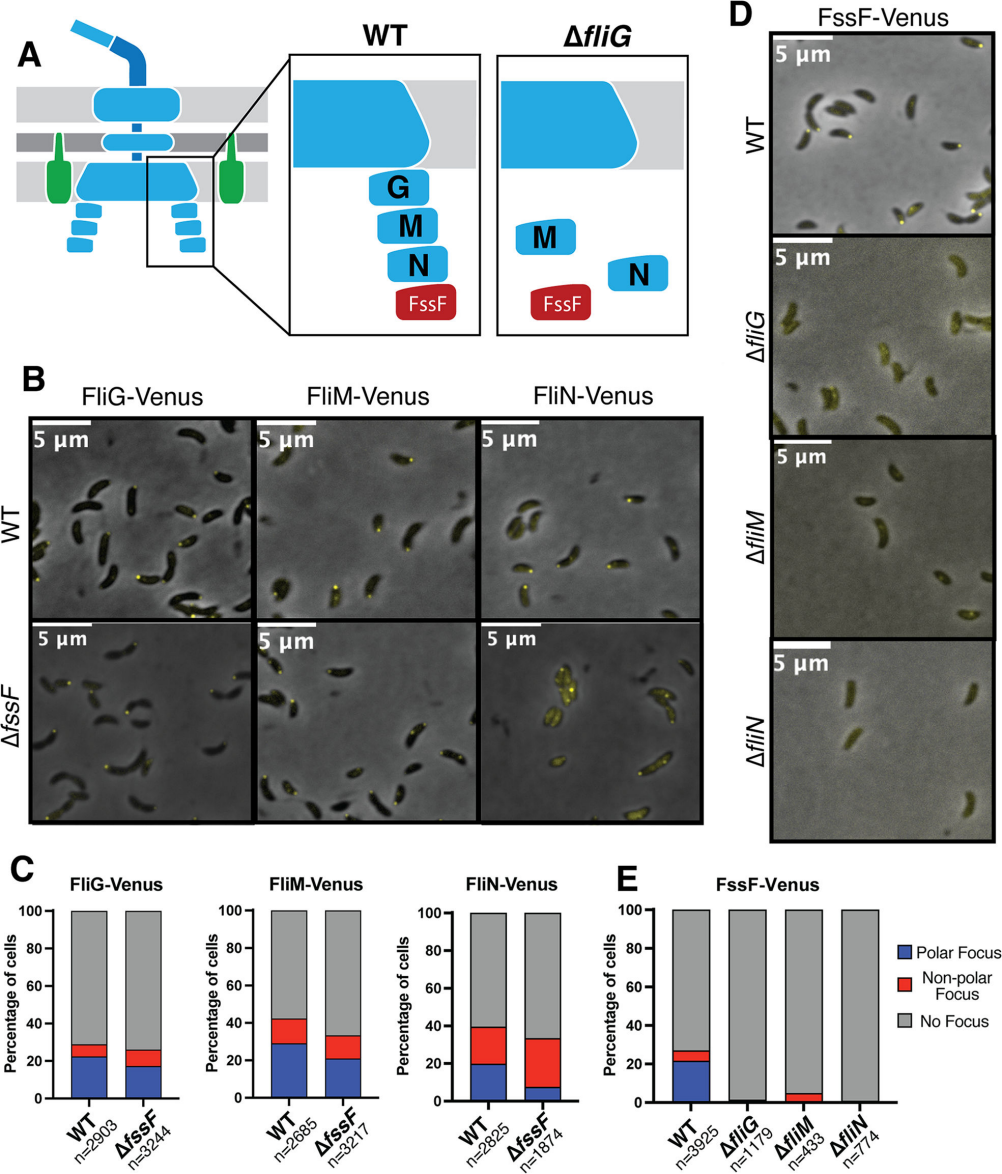
FssF requires the C-Ring for proper localization (**A**) Model of hierarchical assembly of the C-Ring. FliG (**G**) must bind first, followed by FliM (**M**) and FliN (**N**). In the absence of FliG, localization of other C-ring proteins is lost. (**B**) Representative micrographs showing the subcellular localization of FliG-Venus, FliM-Venus, and FliN-Venus in a wild-type (WT) and ∆*fssF* background. (**C**) Quantification of localization of FliG-Venus, FliM-Venus, and FliN-Venus in a wild-type background and ∆*fssF* backround. Cells were binned into three categories based on localization: polar focus, nonpolar focus, and no focus. (**D**) Representative micrographs showing the subcellular localization pattern of FssF-Venus in wild-type (WT), ∆*fliG*, ∆*fliM,* and ∆*fliN* backgrounds. (**E**) Quantification of localization of FssF-Venus in wild-type, ∆*fliG*, ∆*fliM*, and ∆*fliN* backgrounds.

The C-Ring complex in most bacteria is composed of three proteins: FliG, FliM, and FliN (41) (Fig. 1). The C-Ring proteins in *E. coli* bind in a hierarchical manner, meaning that FliG must assemble first to create a scaffold for FliM and FliN to bind (16). Thus, deletion of FliG is predicted to cause mislocalization of all downstream C-Ring components (Fig. 2A). We confirmed this model in *C. cresentus* by fluorescently labeling all three C-Ring proteins (Fig. 2B; Fig. S3). We generated C-terminally tagged forms of each C-Ring gene by fusing *venus* to the 3’ end of each gene at its native locus. The tagged proteins supported wild-type levels of migration through soft agar, and Western blotting showed that the tagged proteins accumulated in their full-length forms (Fig. S2). We performed fluorescence microscopy and observed three types of localization patterns: polar focus, nonpolar focus, and no focus. Wild-type strains containing FliG-Venus, FliM-Venus, or FliN-Venus contained foci specifically at the pole in approximately 20% of cells (Fig. 2B; Fig. S3). Because we utilized unsynchronized populations of *C. crescentus*, a significant portion of the cells are in the stalked phase and do not contain a flagellum. Polar foci for tagged forms of FliG, FliM, and FliN were observed at the pole opposite the stalked pole in predivisional cells, confirming that the polar localization pattern corresponds to the flagellated pole of the cells. In a ∆*fliG* background, both FliM-Venus and FliN-Venus did not display polar foci, indicating mislocalization of these proteins (Fig. S3). In a ∆*fliM* background, FliG-Venus localized to the cell pole, while the strain containing FliN-Venus contained no foci (Fig. S3). In a ∆*fliN* background*,* FliG-Venus exhibited polar localization, while FliM-Venus did not contain foci (Fig. S3). These results are consistent with those of previous literature that indicate FliG is required for localization of downstream C-Ring proteins and that FliN and FliM associate with one another for assembly (16).

To determine whether FssF localizes to the flagellum, we fluorescently tagged *fssF* at its native locus with *Venus* and confirmed the functionality of this fusion using soft agar motility assays and Western blots (Fig. S2). FssF-Venus displays a localization pattern that is indistinguishable from that of FliG, FliM, and FliN in wild-type cells, indicating the protein localizes to the flagellar cell pole (Fig. 2). To confirm its subcellular localization, we localized FssF-Venus in a strain containing an allele of the major flagellin gene (*fljK*) that can be stained with a fluorescent dye. The polar FssF-Venus foci localized to the same pole as the stained flagellar filaments (Fig. S2B). FssF-Venus displayed a diffuse localization pattern when *fliG, fliM,* or *fliN* was deleted (Fig. 2D and E). We found that in a wild-type background, 22% of cells contained a polar FssF-Venus focus, while 0% of cells contained a polar focus in any of the three C-Ring deletion backgrounds (Fig. 2E). These results indicate that FssF requires a properly assembled C-ring for recruitment to the flagellar pole. Deletion of *fssF* did not affect the localization of FliG-Venus or FliM-Venus but increased the percentage of mislocalized FliN-Venus foci, suggesting that *fssF* may affect FliN localization (Fig. 2C).

### FssF is a dispensable for motility, but required for chemotaxis

**Fig 3:**
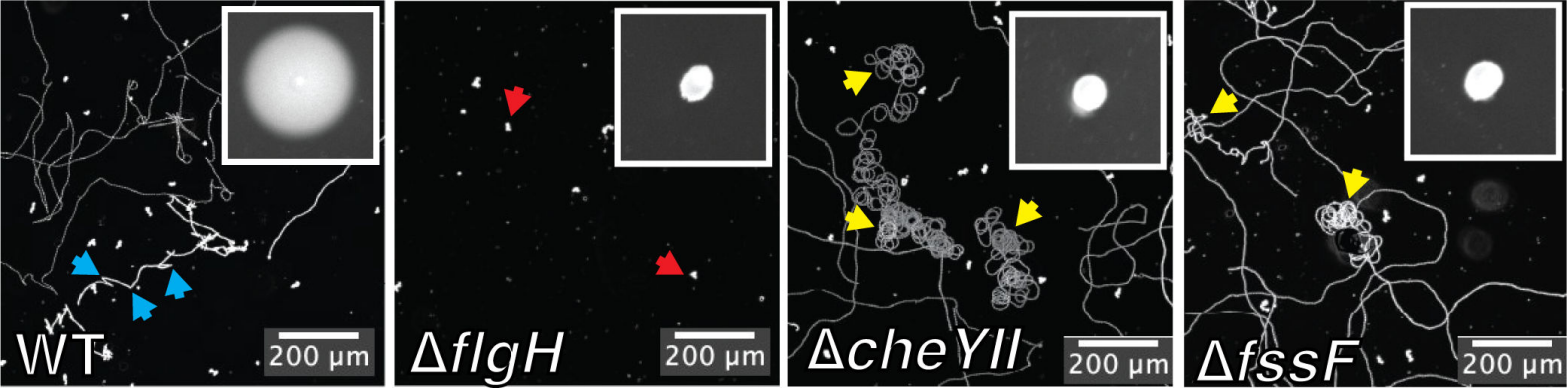
FssF is required for chemotaxis and phenocopies a chemotaxis null mutant in swimming behavior. Maximum projections from live cell tracking of each strain. Immobile cells appear as white spots (red arrows), and swimming trajectories appear as white lines. Directional switching is indicated by blue arrows, while cells unable to change direction are labeled by the yellow arrows. A representative soft agar motility assay is shown in the top right corner of each panel.

To understand the function of *fssF*, we examined the motility phenotypes of the ∆*fssF* mutant using soft agar assays and live cell imaging. ∆*fssF* cells were unable to spread through semi-solid agar (Fig. 3). Spreading in soft agar requires flagellar assembly, motor rotation, and chemotaxis (42). To determine which of these processes was disrupted in the ∆*fssF* mutant, we observed the cellular motility in the liquid medium using live cell imaging. We compared the swimming phenotype in ∆*fssF* to the phenotypes of three other strains: wild-type, ∆*flgH,* and ∆*cheYII*. Wild-type *C. crescentus* cells swim and change directions using the previously described run–reverse–flick sequence (19, 20), while ∆*flgH* cells are nonmotile. ∆*cheYII* cells swim in liquid but do not display run–reverse–flick directional switching and often display spiraling trajectories indicative of being trapped in the hydrodynamic boundary (43). We found that the swimming behavior of ∆*fssF* was indistinguishable from that of the ∆*cheYII* mutant as these cells are capable of swimming but lack directional switching (Fig. 3). We conclude that the ∆*fssF* mutant does not migrate in soft agar due to an inability to perform chemotaxis.

### *fssF* contributes to the stator branch of the *C. crescentus* surface sensing pathway

**Fig 4:**
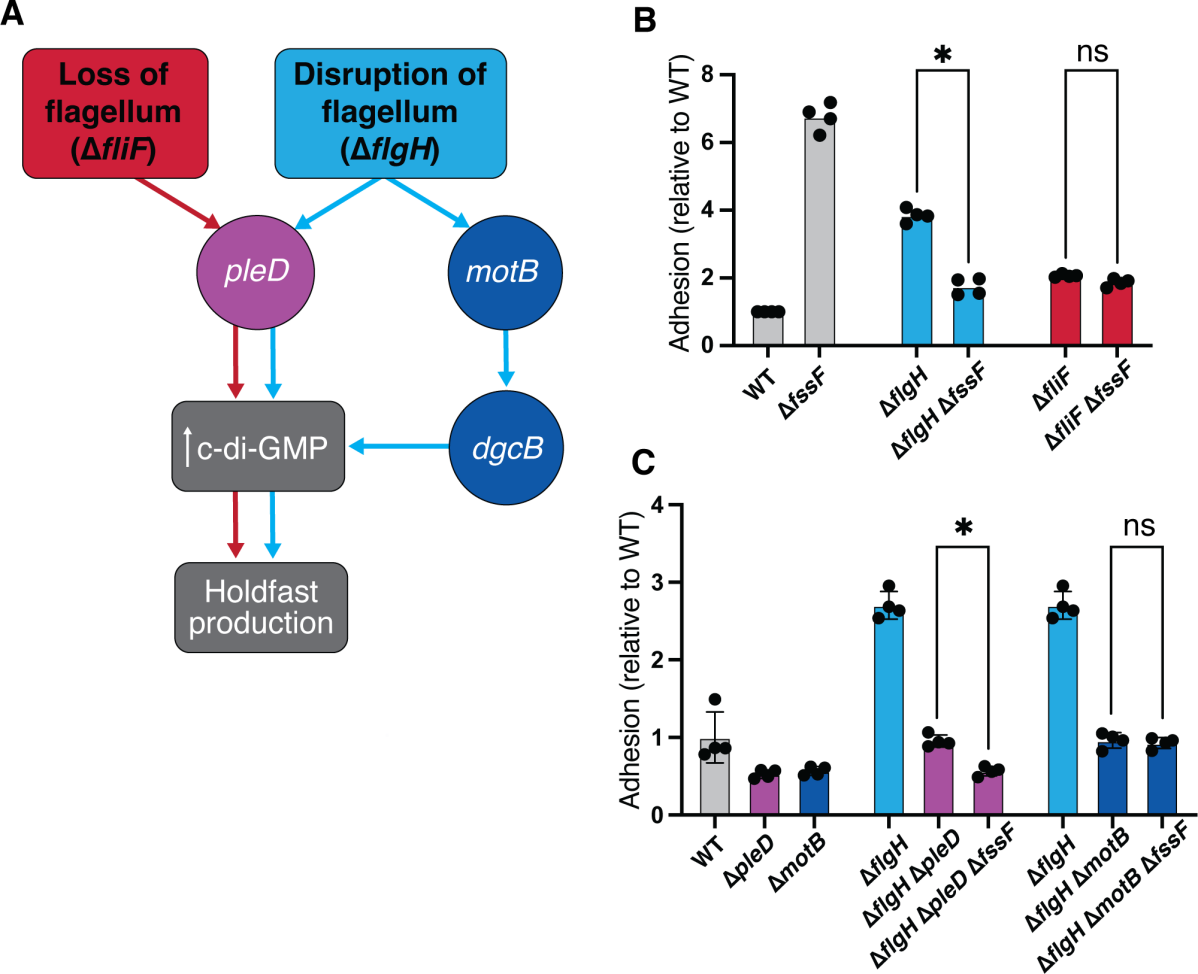
*fssF* is required for full activation of the *motB-*dependent adhesion pathway. (**A**) Model of hyperadhesion regulation induced by loss or disruption of the flagellar machinery. When the flagellum is lost due to mutations in class II flagellar genes such as *fliF* or the C-Ring genes, the *pleD*-dependent adhesion pathway is stimulated. Deletions in genes in the *motB*-dependent adhesion pathway do not affect hyperadhesion in class II flagellar gene mutations. The deletion of class III and IV flagellar genes such as *flgH* results in a hyperadhesion phenotype that is dependent on both *pleD* and *motB*. (**B**) and (**C**) CV staining to measure the adhesion relative to the wild-type. Statistical significance determined by one-way ANOVA test and Tukey’s multiple-comparison test. The asterisk indicates *P*-value of 0.05 or less. The results demonstrate that *fssF* supports ∆*flgH-*dependent hyperadhesion through the mechanical pathway.

We previously determined that late-stage flagellar mutations such as ∆*flgH* activate adhesion by mimicking surface contact and stimulating two distinct signaling pathways (Fig. 4A) (28). To determine if deletion of *fssF* disrupts the developmental or the mechanical pathway, we deleted *fssF* in an early-stage (∆*fliF*) and a late-stage (∆*flgH*) flagellar mutant background. If *fssF* were involved in the developmental pathway, we would expect deletion of *fssF* to suppress hyperadhesion in both the ∆*flgH* and ∆*fliF* backgrounds. If *fssF* were involved in the mechanical pathway, we would expect ∆*fssF* to suppress only ∆*flgH*-mediated hyperadhesion. We used crystal violet (CV) staining to perform these epistasis experiments (Fig. 4B). There was no difference in adhesion between the ∆*fliF* mutant and the double ∆*fliF* ∆*fssF* mutant. However, hyperadhesion in the ∆*flgH* strain was significantly reduced by the deletion of *fssF* (Fig. 4B). These results indicate that *fssF* controls adhesion by supporting the *motB*-dependent, mechanical pathway.

To confirm that *fssF* is required for signaling through the mechanical pathway, we deleted *fssF* in a ∆*flgH* ∆*pleD* background and a ∆*flgH* ∆*motB* background. We expected the ∆*pleD* and ∆*fssF* mutations to have additive effects in suppressing hyperadhesion in the ∆*flgH* background, confirming that these two genes promote hyperedhesion through distinct pathways. Indeed, a ∆*fssF* ∆*flgH* ∆*pleD* triple mutant displayed lower adhesion than the ∆*flgH* ∆*pleD* strain. Deletion of *fssF* did not affect adhesion in the ∆*flgH*∆*motB* background, indicating that *fssF* and *motB* promote adhesion through the same pathway in the ∆*flgH* background (Fig. 4C).

### Disruption of chemotaxis produces a novel hyperadhesive phenotype

**Fig 5:**
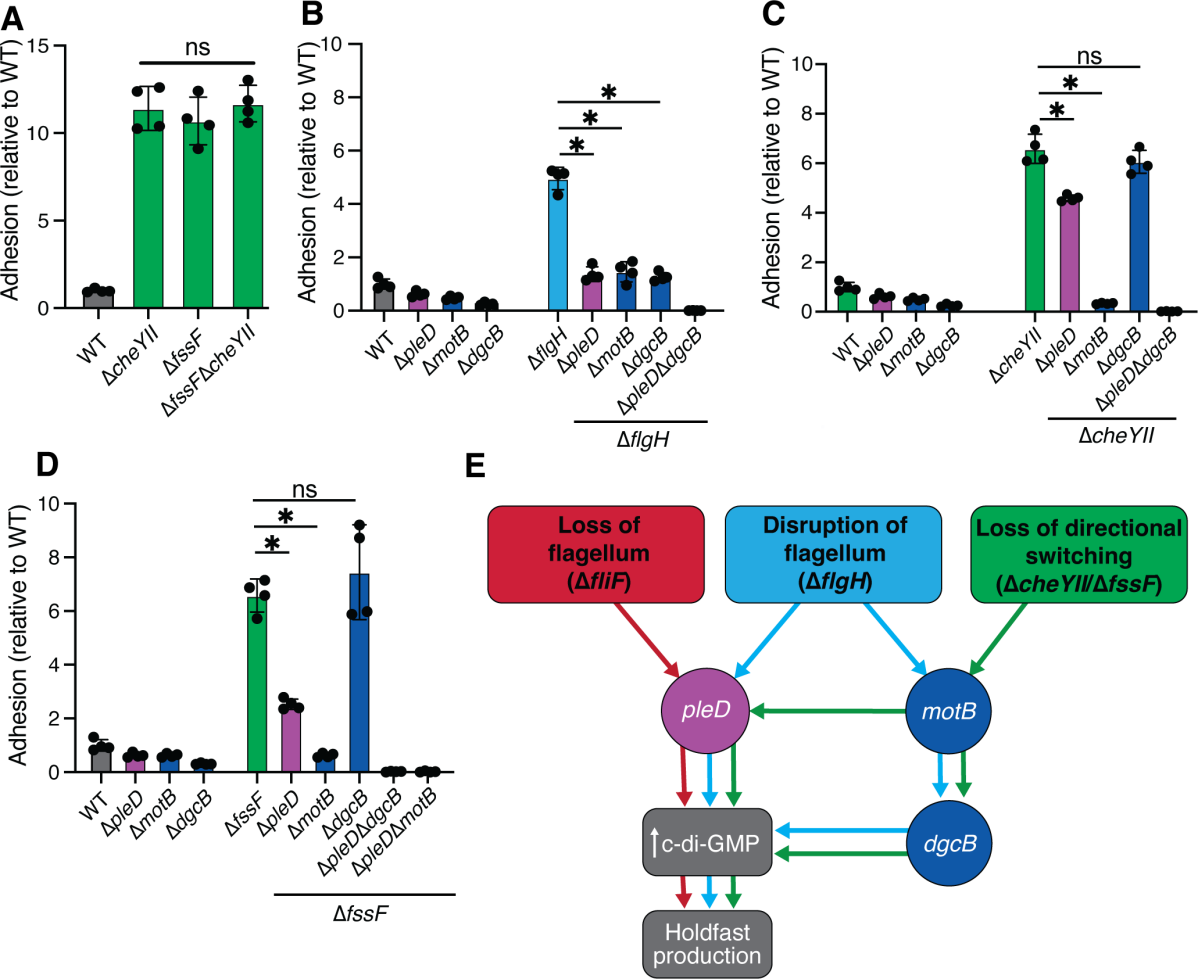
*∆fssF* hyperadhesion differs from ∆*flgH* hyperadhesion. In panels A–D, CV staining comparing adhesion in various mutants to wild-type is shown. (**A**) Che– mutants show a hyperadhesive phenotype. (**B**) Deleting *pleD, motB,* or *dgcB* in the ∆*flgH* mutant background modestly suppresses the hyperadhesvie phenotype. A ∆*flgH* ∆*pleD* ∆*dgcB* triple mutant is nonadhesive, showing that *dgcB* and *pleD* play separate, additive roles in activating adhesion when the flagellar filament is disrupted. (**C and D**) In Che– backgrounds (∆*fssF* and ∆*cheYII* are shown), deleting *pleD* causes minimal suppression of the hyperadhesive phenotype. *motB* is required for adhesion in Che– mutants, while *dgcB* is dispensable. ∆*cheYII* ∆*pleD* ∆*dgcB* and ∆*fssF* ∆*pleD* ∆*dgcB* triple mutants are nonadhesive, showing that *dgcB* and *pleD* play redundant roles in activating adhesion when chemotaxis is disrupted. (**A, B, C, D**) Statistical significance was determined by one-way ANOVA test and Tukey’s multiple-comparison test. The asterisk indicates *P*-value of 0.05 or less. (**E**) Updated model of adhesion regulation incorporating chemotaxis null hyperadhesion. In Che– strains, we find that *pleD* and motB play an important role and that *motB* activates both *pleD* and *dgcB*.

While performing the epistasis experiments described above, we observed that the ∆*fssF* mutation stimulated adhesion in the wild-type background (Fig. 4B). This phenotype is unexpected for suppressors of ∆*flgH* hyperadhesion. These genes are thought to support elevated adhesion when surface sensing is activated, and deleting these genes in the wild-type background typically either does not affect or lowers adhesion (28, 44). We predicted that the ∆*fssF* strain showed elevated adhesion because disrupting chemotaxis increases holdfast production (Fig. S5A). Because both ∆*fssF* and ∆*cheYII* show a Che– motility phenotype, we compared adhesion in the ∆*fssF*, ∆*cheYII,* and double ∆*fssF* ∆*cheYII* mutants using CV staining and direct microscopic quantification of holdfast production (Fig. 5A; Fig. S5A). All three strains exhibited identical hyperadhesive phenotypes, demonstrating that abolishing chemotaxis in *C. crescentus* increases adhesion. To ensure that this phenotype was due to disruption of chemotaxis and not ∆*cheYII-*specific, we also measured adhesion in ∆*cheAI* and found that this strain also displayed a hyperadhesion phenotype (Fig. S5B through D). The ∆*fssF* hyperadhesion phenotype and motility defect could be complemented by expressing the gene from its native promoter at the xylose locus (Fig. S2 and S4).

We sought to understand if regulators of ∆*flgH* hyperadhesion (*pleD, motB,* and *dgcB*) are also involved in the ∆*fssF*/∆*cheYII* hyperadhesion and performed additional epistatsis experiments. As previously described, both *pleD* and *motB* contribute to adhesion in the ∆*flgH* mutant (28). The effects of the ∆*dgcB* mutation were identical to those of the ∆*motB* mutation in the ∆*flgH* background. A ∆*flgH* ∆*pleD* ∆*dgcB* triple mutant is nonadhesive (Fig. 5B), showing that *pleD* and *dgcB* promote adhesion through separate, additive pathways in the ∆*flgH* background.

Deletion of *motB* in the ∆*fssF* and ∆*cheYII* backgrounds nearly abolished adhesion, while deletion of *dgcB* had no effect (Fig. 5C and D). Deletion of *pleD* weakly suppresses hyperadhesion in ∆*cheYII* and ∆*fssF*. Though *dgcB* and *pleD* deletions do not show strong suppression of hyperadhesion individually, deleting both genes simultaneously in the ∆*cheYII*/∆*fssF* backgrounds abolishes adhesion entirely. This indicates that *pleD* and *dgcB* play redundant functions in activating adhesion when chemotaxis is disrupted. We also measured the adhesion of ∆*pleD*, ∆*dgcB*, and ∆*motB* in the ∆*cheAI* background, and the results phenocopied our findings for ∆*cheYII* (Fig. S5B and C). These data suggest that inducing hyperadhesion by disrupting chemotaxis activates a signal transduction sequence that is distinct from the one required for ∆*flgH* hyperadhesion, though the key genes involved remain the same.

## DISCUSSION

Bacteria have robust mechanisms for colonizing solid surfaces, and flagella play crucial roles throughout the colonization sequence (6). Obstruction of the flagellar filament increases recruitment of the stators, which is thought to trigger surface adaptation processes such as adhesion and biofilm formation (27, 38, 45–47). Signal transduction pathways activated by obstruction of the flagellum stimulate the production of the second messenger c-di-GMP, but a more thorough understanding of how the downstream signaling networks are organized is needed. In this study, we characterized a novel surface sensing protein called FssF from the freshwater bacterium *Caulobacter crescentus. fssF* was identified in a screen for signaling factors that activate holdfast production downstream of the flagellum (28), and its predicted protein sequence shows high homology to the C-Ring protein FliN. We found that fluorescently tagged FssF localizes to the flagellar pole of *C. crescentus* cells in a manner dependent on all of the canonical C-Ring proteins (FliG, FliM, and FliN). While disrupting canonical C-Ring genes such as *fliN* abolishes motility (Fig. S2), ∆*fssF* cells retain the ability to swim in liquid but do not perform the directional switching that is characteristic of chemotaxis. Swimming behavior in ∆*fssF* cells is indistinguishable from an established chemotaxis mutant (∆*cheYII*), confirming that FssF is required for chemotaxis.

The requirement of *fssF* for directional switching and the C-Ring-dependent localization of FssF-Venus to the cellular pole support the model that FssF is an accessory protein that associates with the C-Ring of the flagellum. Diversifications and expansions of C-Ring protein repertoires have been identified in other bacteria (13). Our data suggest that duplication of the *fliN* gene led to subfunctionalization of FliN in *C. crescentus* by separating the function of the flagellar assembly from the function of directional switching. FliG, FliM, and FliN are required for assembly of the core C-Ring structure, while FssF is specifically required for chemotaxis. We speculate that either FssF itself or a site on an FssF-dependent C-Ring conformation serves as the binding site for the major CheY-like protein (CheYII) in *C. crescentus*.

Current models for surface sensing emphasize the flagellar motor’s ability to act as a mechanosensor by recruiting additional stator subunits as load on the filament increases (46, 47). Our previous work supported this model by showing that the stator genes are required for the increase in holdfast production when the surface sensing pathway is activated (28). We also identified a second stator-independent pathway activated by disruption of the flagellum, which required the developmental regulator *pleD* (Fig. 4A). Here, we used epistasis analysis to show that *fssF* contributes specifically to the stator-dependent, mechanical branch of the *C. crescentus* surface-sensing network. Given that *fssF* is required for directional switching during chemotaxis (Fig. 3), our results indicate that a robust surface response requires both load-dependent mechanosensing and directional switching. Recent studies from the Sourjik lab provide evidence that deletion of *cheY* in *E. coli* results in decreased biofilm formation in coculture and in the murine gut (48, 49). Though seemingly contradictory to our findings in *C. crescentus* that deletion of the chemotaxis machinery enhances holdfast production, both studies support a common finding that chemotaxis plays a substantive role in regulating biofilm formation.

We also identified a role for chemotaxis genes that is distinct from their function in the mechanical sensing pathway activated by deletion of *flgH*. Mutations disrupting chemotaxis (∆*fssF,* ∆*cheYII,* and ∆*cheAI*) stimulate holdfast production in the wild-type background (Fig. 5A). Berne and Brun reported that a ∆*cheAI* mutant in *C. crescentus* shows a modest decrease in holdfast production (50). While we cannot explain the discrepancy in adhesion phenotypes of the two *C. crescentus* ∆*cheAI* mutants, we have confidence that our Che– mutants show the proper phenotypes. Each of our Che– mutants displays the expected non-spreading phenotype in soft agar (Fig. 3) (42, 51), and Berne and Brun recently reported a genetic screen that identified a hyperadhesive phenotype in a *cheAI* mutant (52). Furthermore, our dissection of the genetic basis for hyperadhesion and confirmation via counting holdfast (Fig. S5) provides additional support for our assignment of holdfast phenotypes.

We used epistasis analysis to show that the ∆*flgH* and ∆*fssF* mutations activate holdfast production differently. In Che– (∆*fssF,* ∆*cheYII,* and ∆*cheAI*) backgrounds, deleting *pleD* causes a slight reduction of the hyperadhesive phenotype, and deleting *dgcB* has no effect. However, the ∆*fssF* ∆*pleD* ∆*dgcB* and ∆*cheYII* ∆*pleD* ∆*dgcB* triple mutants are completely nonadhesive. These results indicate that the two DGCs play redundant roles in activating adhesion when chemotaxis is disrupted. Furthermore, we find that deletion of *motB* nearly abolishes adhesion in the Che– backgrounds. These data indicate that *motB* is upstream of both DGC enzymes. The relationships between these genes differ when hyperadhesion is stimulated by deleting *flgH*. Deleting *motB, pleD,* or *dgcB* in the ∆*flgH* background reduces adhesion to the wild-type level. ∆*flgH* ∆*pleD* ∆*dcgB* and ∆*flgH* ∆*pleD* ∆*motB* triple mutants are completely nonadhesive, but the ∆*flgH* ∆*dgcB* ∆*motB* mutant retains wild-type levels of adhesion. Thus, *pleD* and *dgcB* play separate, additive roles in activating adhesion when late stages of flagellar assembly are disrupted, and *motB* is upstream of *dgcB* but not *pleD*. The separation of the mechanical and developmental pathways is a key difference between adhesion regulation in Fla– mutants compared to Che– strains.

We propose that *pleD, motB,* and *dgcB* and the downstream adhesion factors they control can be activated by multiple stimuli. Though all three genes appear central to a surface sensing network in *C. crescentus*, different sensory inputs seem to activate these genes in different ways. Disrupting the late stages of flagellar assembly activates PleD and DgcB separately, causing these DGCs to perform separate, additive functions. Only DgcB is dependent on the stators in this context. Disrupting directional switching also activates both PleD and DgcB. However, these enzymes play redundant roles in activating adhesion, and both appear dependent on the stators. We propose that the different epistasis patterns observed when holdfast production is activated by disrupting the flagellar assembly or disrupting chemotaxis can be explained by network plasticity. The system appears to alter how signals are transduced in response to different stimuli.

Though we have not yet identified all genes involved in the surface sensing network, it is clear that the stators play a crucial role in directing flux through the system. Eliminating directional switching activates the stators to increase c-di-GMP production by stimulating *pleD* and *dgcB*. Disrupting late stages of flagellar assembly activates *dgcB* via *motB* and activates *pleD* through a separate mechanism (28, 34). Perhaps, ongoing debates surrounding the role(s) of stators in surface sensing can be explained by the fact that they signal differently in different contexts. Understanding how the stators direct flux through the surface sensing network will require characterizing additional genes required for stator-dependent (Che–) and stator-independent (Fla–) activation of *pleD*.

Our work emphasizes that the flagellar motor can integrate diverse sensory stimuli. Mechanical cues from the flagellar filament and chemical cues from chemotaxis systems are integrated into a single response. Recent studies have shown that mechanosensitive stator recruitment increases the affinity of activated CheY for the C-Ring (53). Our analysis indicates that the connection between mechanosensing and directional switching may be even more sophisticated. Not only can load affect CheY binding to the C-Ring, but CheY binding seems to also amplify stator-dependent responses to high load. These findings emphasize the role of the flagellum in environmental sensing. In addition to promoting cellular motility, this machine also serves as a sophisticated signaling hub that allows bacteria to process diverse sensory stimuli.

## MATERIALS AND METHODS

### Bacterial growth and strain constructions

*C. crescentus* CB15 cells were grown in peptone yeast extract (PYE) broth by shaking at 200 rpm overnight at 30 ˚C, unless otherwise indicated. Plasmids for deleting individual genes were generated by amplifying ~500 bp upstream and downstream of the target gene along with a few (usually four) codons from the start and end of the ORF. These fragments were fused and inserted into pNTPS138 using Gibson assembly (54) to generate a suicide plasmid for removing the central portion of the target gene. Complementation of mutants was achieved by inserting the target gene into pXGFPC-2 (55) along with its native promoter. Plasmids for inserting tagged alleles of various genes were generated by amplifying ~500 bp upstream of the top codon for the target ORF, the *Venus* tag from pXVENC-2, and ~500 bp downstream of the stop codon for the target ORF. These three fragments were assembled into pNTPS138 using Gibson assembly to generate a plasmid that replaces the target gene with a C-terminally *Venus-*tagged allele. Detailed descriptions of each construct can be found in Table S2. Gibson assemblies were transformed into DH5 α *Escherichia coli-*competent cells via heat shock. *E. coli* cultures were grown in Luria–Bertani (LB) broth supplemented with 50 ug/mL kanamycin when required. Deletion and insertion plasmids were transformed into *C. crescentus* CB15 through electroporation, followed by kanamycin selection (25 ug/mL kanamycin) and a sacB-based counter-selection using 3% wt/vol sucrose (56). Strains are listed in Table S1 and plasmids in Table S2. Primer and plasmids sequences are available upon request.

### Crystal violet staining assay

*C. crescentus* cultures were grown overnight in PYE and then diluted to an optical density (OD_660_) of 0.5 with PYE. A 48-well plate was prepared with 450 uL of M2X media (1X M2 salts, 1% hunter base, 0.5 M CaCl2, 1M MgSO4, and 0.15% xylose) in each well (39). Each well was inoculated with 1.5 uL of the diluted culture. Each plate contained four replicates. Plates were sealed and grown at 30 ˚C while shaking at 155 rpm for 17 hours. Cultures were then discarded, and the plates were washed continuously under running cold tap water for 1–2 minutes. Remaining adherent cells were stained by adding 500 uL of 0.01% crystal violet aqueous solution to each well and shaking the plates at room temperature for 10  minutes. The plates were washed for 1–2 minutes with running tap water. The remaining dye was dissolved by adding 500 uL of ethanol to each well and shaking for another 10 minutes. Staining was quantified by measuring the absorbance at 575  nm using a BioTek Synergy H1microplate reader. The absorbance value of each reading was first subtracted by the absorbance value of a blank sample. These values were then normalized and compared to the average WT absorbance value. Statistical analysis was performed through GraphPad Prism.

### Soft agar assay

*C. crescentus* PYE cultures were grown overnight and then diluted to an OD_660_ of 0.5 with PYE. Approximately 1.5 uL of diluted cultures was inoculated in PYE plates containing 0.2% agar. The plates were then left to incubate at 30°C for 72–96 hours. Images were taken using the Invitrogen iBright FL1500 Imaging System.

### Live cell microscopy to analyze swimming behavior

PYE overnight cultures of 2 mL were backdiluted to an OD_660_ of 0.1. The cells were then grown to OD_660_ of 0.4–0.5 and diluted 1,000-fold in a microfuge tube containing 1 mL of PYE. Glass slides were inoculated with 2 uL of cells and enclosed with a coverslip and sealed with valap. Dark-field images were taken with a 10 x objective lens every 50 msec for 1 minute in a Nikon Eclipse Ti series microscope and Orca Fusion BT digital CMOS camera (Hamamatsu). Maximum projections were calculated using Nikon NIS Elements software.

### Imaging fluorescently tagged C-Ring proteins

Overnight PYE cultures of *C. crescentus* were backdiluted in M6HIGX (5 mM imidazole-HCl (pH 7.0), 2 mM sodium phosphate, 1% hunter base, 0.3% sodium glutamate-KOH (pH 7.0), and 0.3% xylose) to an OD660 of 0.1. At an OD660 of 0.4–0.5, 100 uL of the culture was collected in a microcentrifuge tube and spun-down for 1 minute at 3,500 rcf. Then, 85 uL of the supernatant was removed, and the remaining pellet was resuspended by agitating the tube gently. Two microliters of the concentrated culture was plated on a 3% agarose pad. A Nikon Eclipse Ti series microscope with a 100 x oil immersion objective lens was used to capture the fluorescently tagged proteins in cells. Fluorescence images were collected using a Prior Lumen 200 metal halide light source and a YFP-specific filter set (Chroma). Images were taken with 25–50 msec phase exposure and 3-second fluorescence exposure. All images shown were processed using ImageJ, and microbeJ was used for identification of cells and polar foci.

### Holdfast staining

*C. crescentus* cultures were grown overnight in PYE, then diluted 1:400 in M2X medium, and grown overnight to an OD_660_ between 0.05 and 0.1. At the target OD, a sample of culture was taken, and Alexa 594-WGA was added to a final concentration of 2 ng/mL. The sample was then washed with 1 mL of sterile H_2_O and centrifuged for 2 minutes at 6K x *g*. The supernatant was removed, and cells were resuspended by agitating the tube gently. Two microliters of the sample was added to 3% agarose pad and imaged on a Nikon Eclipse Ti series microscope with a 100 x oil immersion objective lens with a 50 msec phase exposure, followed by a 1–2 sec mCherry exposure. Fluorescence images were collected using a Prior Lumen 200 metal halide light source and a YFP- and mCherry-specific filter set (Chroma). All images shown were processed using ImageJ and microbeJ (57).

### Immunoblotting

Cells were grown overnight in PYE and then backdiluted to an OD660 of 0.1. Cells from 0.5 mL of the culture (OD_660_ of 0.4–0.5) were collected by centrifugation. Pellets were resuspended in 1 x TBS (20 mM Tris, 0.15M NaCl, pH 7.5) with benzonase nuclease, incubated at 30°C for 10 minutes, and added to an equal concentration of 4 x SDS-LOAD buffer containing 1% BME. Samples were boiled, loaded into 12% SDS-PAGE gels, resolved, and transferred to a PVDF membrane via semi-dry transfer. Blocking was performed with 5% milk powder for 2 hours at room temperature. The membrane was then shaken overnight at 4°C in 5% milk containing 1:10,000 dilution of anti-GFP serum from rabbit. The membrane was then subjected to three 15-minute washes in 1 X TBST. Anti-rabbit HRP was applied in 5% milk at a concentration of 1:10,000 and shaken at room temperature for 1 hour. Gels were exposed on Invitrogen iBright FL1500 Imaging System using chemiluminescent substrate Western Lightning Plus by PerkinElmer.

### Flagellar staining

Cells were grown overnight in PYE and backdiluted the next morning to an OD_660_ of 0.1 in M6HIGX (5 mM imidazole-HCl (pH 7.0), 2 mM sodium phosphate, 1% hunter base, 0.3% sodium glutamate-KOH (pH 7.0), and 0.3% xylose) and PYE. Cultures were grown to an OD_660_ of 0.4–0.5 at 30°C. Approximately 500 uL of the culture was collected from each growth condition, and 0.5 uL of a 2 mg/mL solution of Alexa Fluor 594 C5-maleimide (Invitrogen) dissolved in dimethyl sulfoxide was added and incubated for 10 minutes in the dark. One milliliter of sterile H2O was added for a wash, and samples were centrifuged at 3,500 x *g* for 2 minutes. The supernatant was poured off, and remaining cells were gently resuspended via flicking. Then, 2 uL of the sample was applied to a 3% agarose pad. A Nikon Eclipse Ti series microscope with a 100 x oil immersion objective lens was used to capture the fluorescently tagged proteins in cells. Fluorescence images were collected using a Prior Lumen 200 metal halide light source and a YFP- and mCherry-specific filter set (Chroma). Images were taken with 25–50 msec phase exposure, a 3–4 s YFP exposure, and a 200–300 ms mCherry exposure.

## SUPPLEMENTAL MATERIAL

Supplemental figures and tables jb.00404-24-s0001.docx Fig. S1 to S5; Tables S1 and S2.

ASM does not own the copyrights to Supplemental Material that may be linked to, or accessed through, an article. The authors have granted ASM a non-exclusive, world-wide license to publish the Supplemental Material files. Please contact the corresponding author directly for reuse.

## References

[1] Dang H, Lovell CR. 2016. Microbial surface colonization and biofilm development in marine environments. Microbiol Mol Biol Rev 80:91–138. doi:10.1128/MMBR.00037-15 26700108 PMC4711185

[2] Harshey RM, Partridge JD. 2015. Shelter in a swarm. J Mol Biol 427:3683–3694. doi:10.1016/j.jmb.2015.07.025 26277623 PMC4548829

[3] O’Toole GA, Wong GC. 2016. Sensational biofilms: surface sensing in bacteria. Curr Opin Microbiol 30:139–146. doi:10.1016/j.mib.2016.02.004 26968016 PMC4843124

[4] O’Toole G, Kaplan HB, Kolter R. 2000. Biofilm formation as microbial development. Annu Rev Microbiol 54:49–79. doi:10.1146/annurev.micro.54.1.49 11018124

[5] Muhammad MH, Idris AL, Fan X, Guo Y, Yu Y, Jin X, Qiu J, Guan X, Huang T. 2020. Beyond risk: bacterial biofilms and their regulating approaches. Front Microbiol 11:928. doi:10.3389/fmicb.2020.00928 32508772 PMC7253578

[6] Belas R. 2014. Biofilms, flagella, and mechanosensing of surfaces by bacteria. Trends Microbiol 22:517–527. doi:10.1016/j.tim.2014.05.002 24894628

[7] Berne C, Ellison CK, Ducret A, Brun YV. 2018. Bacterial adhesion at the single-cell level. Nat Rev Microbiol 16:616–627. doi:10.1038/s41579-018-0057-5 30008468

[8] Haiko J, Westerlund-Wikström B. 2013. The role of the bacterial flagellum in adhesion and virulence. Biology (Basel) 2:1242–1267. doi:10.3390/biology2041242 24833223 PMC4009794

[9] Aldridge P, Hughes KT. 2002. Regulation of flagellar assembly. Curr Opin Microbiol 5:160–165. doi:10.1016/s1369-5274(02)00302-8 11934612

[10] Minamino T, Imada K. 2015. The bacterial flagellar motor and its structural diversity. Trends Microbiol 23:267–274. doi:10.1016/j.tim.2014.12.011 25613993

[11] Wu J, Newton A. 1997. Regulation of the Caulobacter flagellar gene hierarchy; not just for motility. Mol Microbiol 24:233–239. doi:10.1046/j.1365-2958.1997.3281691.x 9159510

[12] Chang Y, Zhang K, Carroll BL, Zhao X, Charon NW, Norris SJ, Motaleb MA, Li C, Liu J. 2020. Molecular mechanism for rotational switching of the bacterial flagellar motor. Nat Struct Mol Biol 27:1041–1047. doi:10.1038/s41594-020-0497-2 32895555 PMC8129871

[13] Henderson LD, Matthews-Palmer TRS, Gulbronson CJ, Ribardo DA, Beeby M, Hendrixson DR. 2020. Diversification of Campylobacter jejuni flagellar C-ring composition impacts its structure and function in motility, flagellar assembly, and cellular processes. MBio 11:e02286-19. doi:10.1128/mBio.02286-19 31911488 PMC6946799

[14] Johnson S, Deme JC, Furlong EJ, Caesar JJE, Chevance FFV, Hughes KT, Lea SM. 2024. Structural basis of directional switching by the bacterial flagellum. Nat Microbiol 9:1282–1292. doi:10.1038/s41564-024-01630-z 38459206

[15] Khan IH, Reese TS, Khan S. 1992. The cytoplasmic component of the bacterial flagellar motor. Proc Natl Acad Sci U S A 89:5956–5960. doi:10.1073/pnas.89.13.5956 1631080 PMC402117

[16] Kubori T, Yamaguchi S, Aizawa S. 1997. Assembly of the switch complex onto the MS ring complex of Salmonella typhimurium does not require any other flagellar proteins. J Bacteriol 179:813–817. doi:10.1128/jb.179.3.813-817.1997 9006037 PMC178764

[17] Zhao R, Pathak N, Jaffe H, Reese TS, Khan S. 1996. FliN is a major structural protein of the C-ring in the Salmonella typhimurium flagellar basal body. J Mol Biol 261:195–208. doi:10.1006/jmbi.1996.0452 8757287

[18] Sridhar A. 2020. The inner workings of the flagellar motor. Nat Rev Microbiol 18:673–673. doi:10.1038/s41579-020-00473-9 33067569

[19] Koyasu S, Shirakihara Y. 1984. Caulobacter crescentus flagellar filament has a right-handed helical form. J Mol Biol 173:125–130. doi:10.1016/0022-2836(84)90407-8 6366238

[20] Liu B, Gulino M, Morse M, Tang JX, Powers TR, Breuer KS. 2014. Helical motion of the cell body enhances Caulobacter crescentus motility. Proc Natl Acad Sci U S A 111:11252–11256. doi:10.1073/pnas.1407636111 25053810 PMC4128131

[21] Bi S, Sourjik V. 2018. Stimulus sensing and signal processing in bacterial chemotaxis. Curr Opin Microbiol 45:22–29. doi:10.1016/j.mib.2018.02.002 29459288

[22] Hansen CH, Endres RG, Wingreen NS. 2008. Chemotaxis in Escherichia coli: a molecular model for robust precise adaptation. PLoS Comput Biol 4:e1. doi:10.1371/journal.pcbi.0040001 18179279 PMC2174977

[23] Huang Z, Pan X, Xu N, Guo M. 2019. Bacterial chemotaxis coupling protein: structure, function and diversity. Microbiol Res 219:40–48. doi:10.1016/j.micres.2018.11.001 30642465

[24] Szurmant H, Ordal GW. 2004. Diversity in chemotaxis mechanisms among the bacteria and archaea. Microbiol Mol Biol Rev 68:301–319. doi:10.1128/MMBR.68.2.301-319.2004 15187186 PMC419924

[25] Muok AR, Briegel A, Crane BR. 2020. Regulation of the chemotaxis histidine kinase CheA: a structural perspective. Biochim Biophys Acta Biomembr 1862:183030. doi:10.1016/j.bbamem.2019.183030 31374212 PMC7212787

[26] Sarkar MK, Paul K, Blair D. 2010. Chemotaxis signaling protein CheY binds to the rotor protein FliN to control the direction of flagellar rotation in Escherichia coli. Proc Natl Acad Sci U S A 107:9370–9375. doi:10.1073/pnas.1000935107 20439729 PMC2889077

[27] McCarter L, Hilmen M, Silverman M. 1988. Flagellar dynamometer controls swarmer cell differentiation of V. parahaemolyticus. Cell 54:345–351. doi:10.1016/0092-8674(88)90197-3 3396074

[28] Hershey DM, Fiebig A, Crosson S. 2021. Flagellar perturbations activate adhesion through two distinct pathways in Caulobacter crescentus. MBio 12:e03266-20. doi:10.1128/mBio.03266-20 33563824 PMC7885107

[29] Ross P, Weinhouse H, Aloni Y, Michaeli D, Weinberger-Ohana P, Mayer R, Braun S, de Vroom E, van der Marel GA, van Boom JH, Benziman M. 1987. Regulation of cellulose synthesis in Acetobacter xylinum by cyclic diguanylic acid. Nature New Biol 325:279–281. doi:10.1038/325279a0 18990795

[30] Hershey DM. 2021. Integrated control of surface adaptation by the bacterial flagellum. Curr Opin Microbiol 61:1–7. doi:10.1016/j.mib.2021.02.004 33640633

[31] Bodenmiller D, Toh E, Brun YV. 2004. Development of surface adhesion in Caulobacter crescentus. J Bacteriol 186:1438–1447. doi:10.1128/JB.186.5.1438-1447.2004 14973013 PMC344395

[32] Schniederberend M, Williams JF, Shine E, Shen C, Jain R, Emonet T, Kazmierczak BI. 2019. Modulation of flagellar rotation in surface-attached bacteria: a pathway for rapid surface-sensing after flagellar attachment. PLoS Pathog 15:e1008149. doi:10.1371/journal.ppat.1008149 31682637 PMC6855561

[33] Ellison CK, Kan J, Dillard RS, Kysela DT, Ducret A, Berne C, Hampton CM, Ke Z, Wright ER, Biais N, Dalia AB, Brun YV. 2017. Obstruction of pilus retraction stimulates bacterial surface sensing. Science 358:535–538. doi:10.1126/science.aan5706 29074778 PMC5805138

[34] Hug I, Deshpande S, Sprecher KS, Pfohl T, Jenal U. 2017. Second messenger-mediated tactile response by a bacterial rotary motor. Science 358:531–534. doi:10.1126/science.aan5353 29074777

[35] Skerker JM, Laub MT. 2004. Cell-cycle progression and the generation of asymmetry in Caulobacter crescentus. Nat Rev Microbiol 2:325–337. doi:10.1038/nrmicro864 15031731

[36] Jenal U. 2000. Signal transduction mechanisms in Caulobacter crescentus development and cell cycle control. FEMS Microbiol Rev 24:177–191. doi:10.1016/S0168-6445(99)00035-2 10717313

[37] Chepkwony NK, Brun YV. 2021. A polysaccharide deacetylase enhances bacterial adhesion in high-ionic-strength environments. iScience 24:103071. doi:10.1016/j.isci.2021.103071 34568792 PMC8449245

[38] Li G, Brown PJB, Tang JX, Xu J, Quardokus EM, Fuqua C, Brun YV. 2012. Surface contact stimulates the just-in-time deployment of bacterial adhesins. Mol Microbiol 83:41–51. doi:10.1111/j.1365-2958.2011.07909.x 22053824 PMC3245333

[39] Hershey DM, Fiebig A, Crosson S. 2019. A genome-wide analysis of adhesion in Caulobacter crescentus identifies new regulatory and biosynthetic components for holdfast assembly. MBio 10:e02273-18. doi:10.1128/mBio.02273-18 30755507 PMC6372794

[40] Bischoff DS, Ordal GW. 1992. Identification and characterization of FliY, a novel component of the Bacillus subtilis flagellar switch complex. Mol Microbiol 6:2715–2723. doi:10.1111/j.1365-2958.1992.tb01448.x 1447979

[41] Chen S, Beeby M, Murphy GE, Leadbetter JR, Hendrixson DR, Briegel A, Li Z, Shi J, Tocheva EI, Müller A, Dobro MJ, Jensen GJ. 2011. Structural diversity of bacterial flagellar motors. EMBO J 30:2972–2981. doi:10.1038/emboj.2011.186 21673657 PMC3160247

[42] Wolfe AJ, Berg HC. 1989. Migration of bacteria in semisolid agar. Proc Natl Acad Sci U S A 86:6973–6977. doi:10.1073/pnas.86.18.6973 2674941 PMC297974

[43] Conrad JC. 2012. Physics of bacterial near-surface motility using flagella and type IV pili: implications for biofilm formation. Res Microbiol 163:619–629. doi:10.1016/j.resmic.2012.10.016 23103335

[44] Hellenbrand CN, Stevenson DM, Gromek KA, Amador-Noguez D, Hershey DM. 2024. A deoxynucleoside triphosphate triphosphohydrolase promotes cell cycle progression in Caulobacter crescentus. bioRxiv:2024.04.25.591158. doi:10.1101/2024.04.25.591158 PMC12186491 40454833

[45] Baker AE, O’Toole GA. 2017. Bacteria, rev your engines: stator dynamics regulate flagellar motility. J Bacteriol 199:e00088-17. doi:10.1128/JB.00088-17 28320878 PMC5446623

[46] Lele PP, Hosu BG, Berg HC. 2013. Dynamics of mechanosensing in the bacterial flagellar motor. Proc Natl Acad Sci U S A 110:11839–11844. doi:10.1073/pnas.1305885110 23818629 PMC3718179

[47] Tipping MJ, Delalez NJ, Lim R, Berry RM, Armitage JP. 2013. Load-dependent assembly of the bacterial flagellar motor. MBio 4:e00551-13. doi:10.1128/mBio.00551-13 23963182 PMC3747592

[48] Laganenka L, Lee J-W, Malfertheiner L, Dieterich CL, Fuchs L, Piel J, von Mering C, Sourjik V, Hardt W-D. 2023. Chemotaxis and autoinducer-2 signalling mediate colonization and contribute to co-existence of Escherichia coli strains in the murine gut. Nat Microbiol 8:204–217. doi:10.1038/s41564-022-01286-7 36624229

[49] Laganenka L, Sourjik V. 2018. Autoinducer 2-dependent Escherichia coli biofilm formation is enhanced in a dual-species coculture. Appl Environ Microbiol 84:e02638-17. doi:10.1128/AEM.02638-17 29269492 PMC5812939

[50] Berne C, Brun YV. 2019. The two chemotaxis clusters in Caulobacter crescentus play different roles in chemotaxis and biofilm regulation. J Bacteriol 201:e00071-19. doi:10.1128/JB.00071-19 31109992 PMC6707910

[51] Skerker JM, Prasol MS, Perchuk BS, Biondi EG, Laub MT. 2005. Two-component signal transduction pathways regulating growth and cell cycle progression in a bacterium: a system-level analysis. PLoS Biol 3:e334. doi:10.1371/journal.pbio.0030334 16176121 PMC1233412

[52] Zappa S, Berne C, Morton Iii RI, Whitfield GB, De Stercke J, Brun YV. 2024. The HmrABCX pathway regulates the transition between motile and sessile lifestyles in Caulobacter crescentus by a mechanism independent of hfiA transcription. MBio:e0100224. doi:10.1128/mbio.01002-24 39230277 PMC11481889

[53] Antani JD, Sumali AX, Lele TP, Lele PP. 2021. Asymmetric random walks reveal that the chemotaxis network modulates flagellar rotational bias in Helicobacter pylori. Elife 10:e63936. doi:10.7554/eLife.63936 33493107 PMC7834020

[54] Gibson DG, Young L, Chuang R-Y, Venter JC, Hutchison CA, Smith HO. 2009. Enzymatic assembly of DNA molecules up to several hundred kilobases. Nat Methods 6:343–345. doi:10.1038/nmeth.1318 19363495

[55] Thanbichler M, Iniesta AA, Shapiro L. 2007. A comprehensive set of plasmids for vanillate- and xylose-inducible gene expression in Caulobacter crescentus. Nucleic Acids Res 35:e137. doi:10.1093/nar/gkm818 17959646 PMC2175322

[56] Hmelo LR, Borlee BR, Almblad H, Love ME, Randall TE, Tseng BS, Lin C, Irie Y, Storek KM, Yang JJ, Siehnel RJ, Howell PL, Singh PK, Tolker-Nielsen T, Parsek MR, Schweizer HP, Harrison JJ. 2015. Precision-engineering the Pseudomonas aeruginosa genome with two-step allelic exchange. Nat Protoc 10:1820–1841. doi:10.1038/nprot.2015.115 26492139 PMC4862005

[57] Ducret A, Quardokus EM, Brun YV. 2016. MicrobeJ, a tool for high throughput bacterial cell detection and quantitative analysis. Nat Microbiol 1:16077. doi:10.1038/nmicrobiol.2016.77 27572972 PMC5010025

