# Supplementary material for: A flagellar accessory protein links chemotaxis to surface sensing
Source: J Bacteriol. 2024 Oct 18;206(11):e00404-24. doi: 10.1128/jb.00404-24 (PMC11580411; doi:10.1128/jb.00404-24)
Supplement: Supplemental figures and tables — Fig. S1 to S5; Tables S1 and S2. [file jb.00404-24-s0001.docx]

Supplemental Data
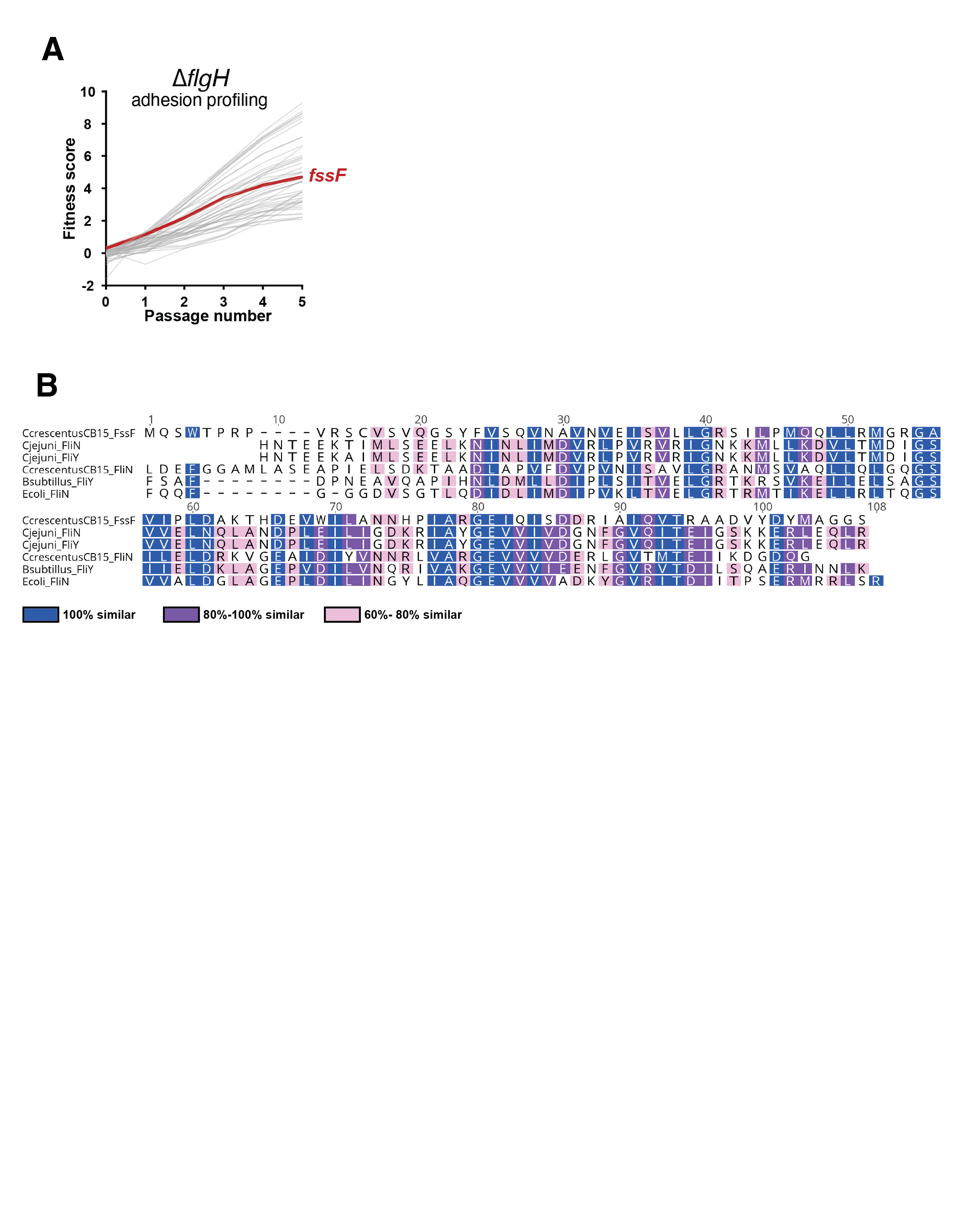


Figure S1: Identification of FliN homologue FssF. (A) Adhesion profiling in a ∆*flgH* background (Hershey et al. 2021). Adhesion profiling utilizes a piece of cheesecloth suspended in liquid medium to sequester adherent bacteria. Each passage is accomplished by inoculating fresh medium containing a cheesecloth with the liquid culture of the previous day. By passaging the liquid culture away from the cheesecloth, we enrich for genes that decrease adhesion when interrupted. Each line represents a different gene that is interrupted by a transposon. The higher the fitness score, the more represented that transposon mutant is in the population. The gene of interest, FssF, is highlighted in red. (B) Alignment of FssF to FliN amino acid sequence from *C. crescentus* and other organisms.


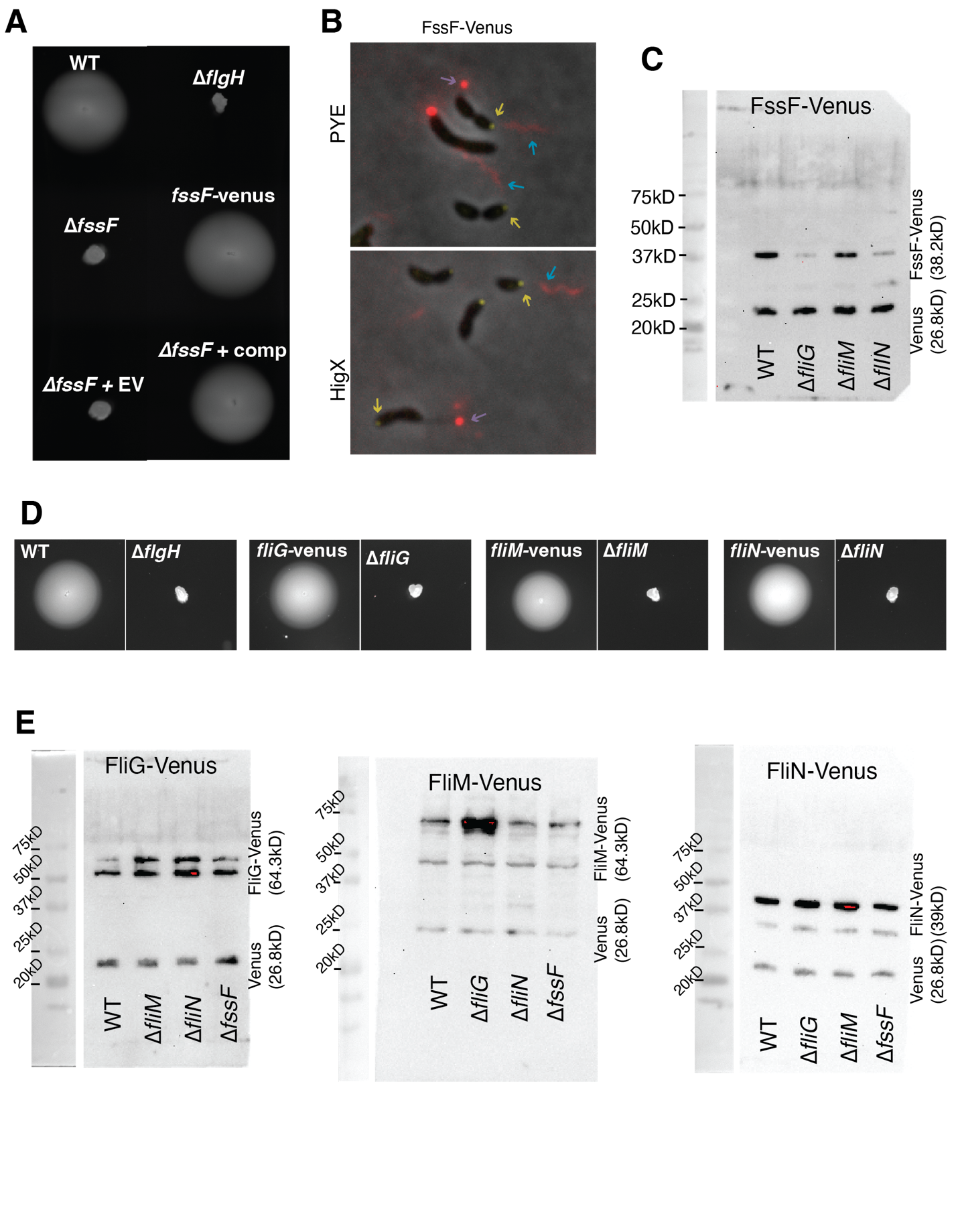


Figure S2: Functionality of *venus* tagged alleles. (A, D) Soft agar assays comparing motility phenotypes of *venus* tagged proteins. ∆*flgH* is used as a non-motile control. (B) Microscopy images of strain harboring FssF-Venus at the native locus and fljK^T103C^ substitution. Flagella are and holdfast are dyed with Alexa Fluor 594 C5-maleimide. Yellow arrows indicate a Fssf-Venus foci, blue arrows indicate a dyed flagellum, and purple indicate a holdfast. (C, E) Western blots using anti-GFP antibody of Venus tagged proteins in all relevant backgrounds. Full-length fusions proteins of the expected size are detected in all backgrounds.


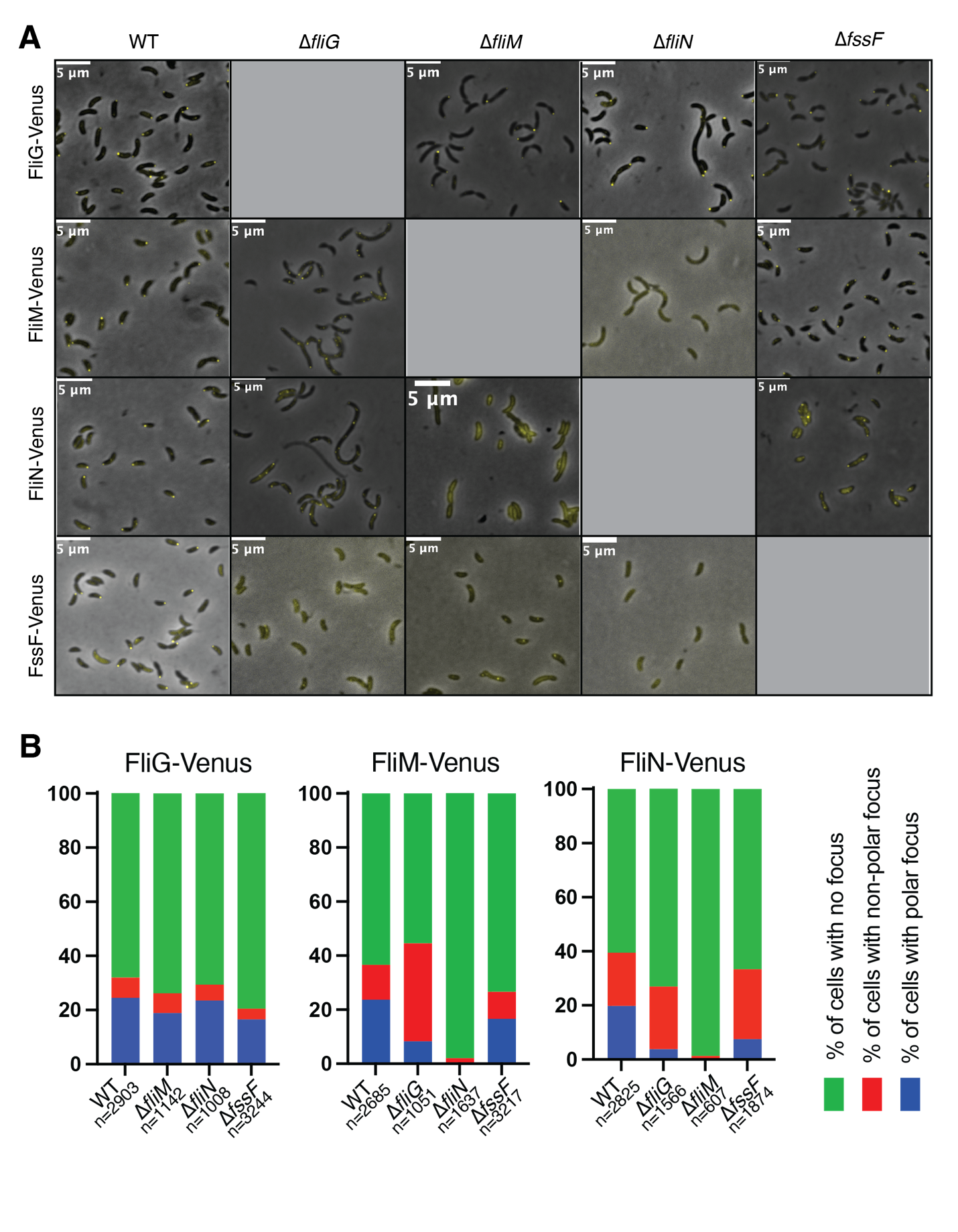


Figure S3: Localization patterns of venus tagged proteins. (A) Representative images of Venus-tagged C-ring proteins and FssF in 5 backgrounds: WT, ∆*fliG*, ∆*fliM*, ∆*fliN*, ∆*fssF.* (B) Quantification of localization patterns in various backgrounds.


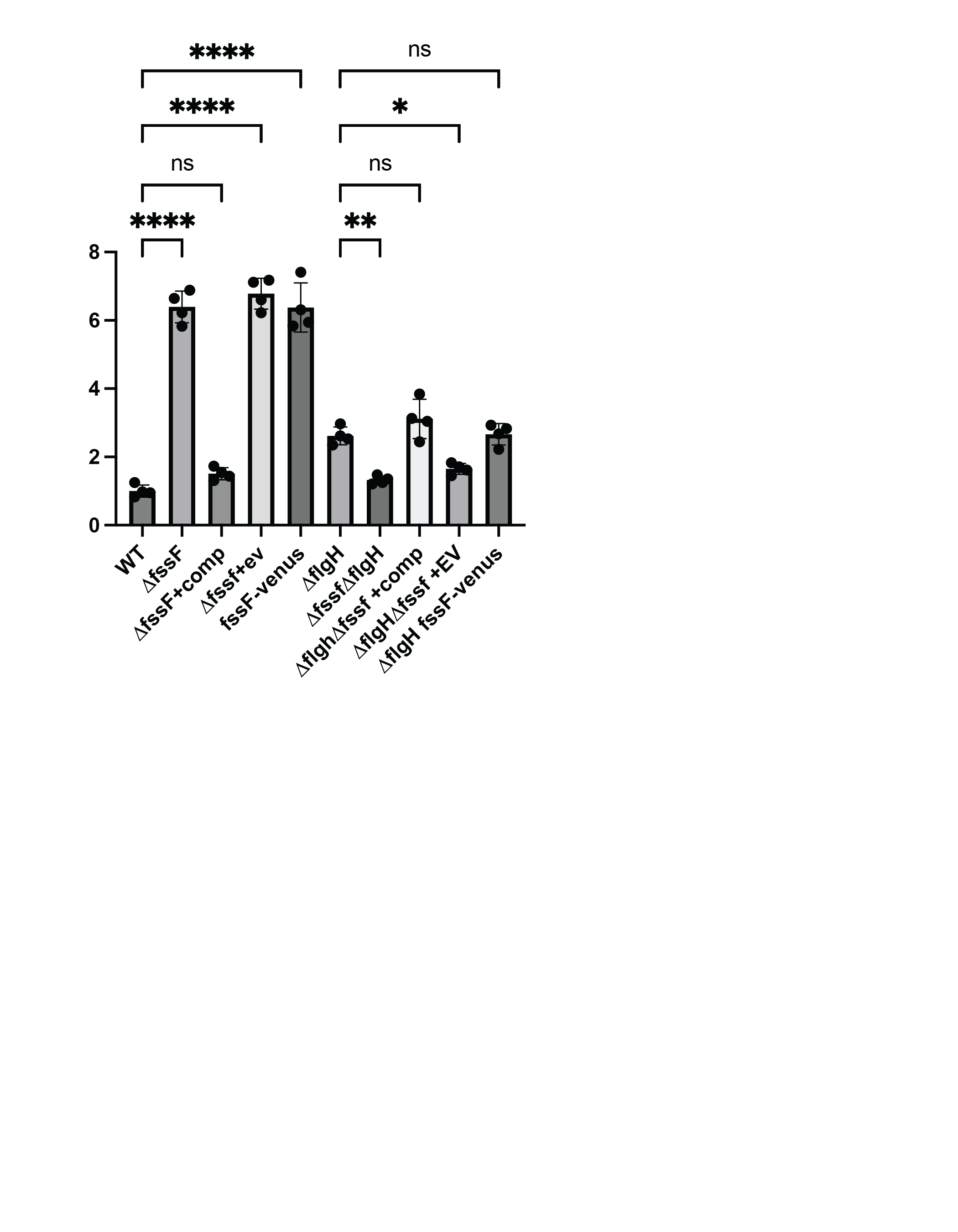


Figure S4: Genetic complementation of ∆*fssF*. (A) CV staining in various backgrounds comparing the complementation, empty vector, and venus tag. Statistical significance was determined by one-way ANOVA test and Tukey’s multiple-comparison test. Asterisk indicates P-value of 0.05 or less.


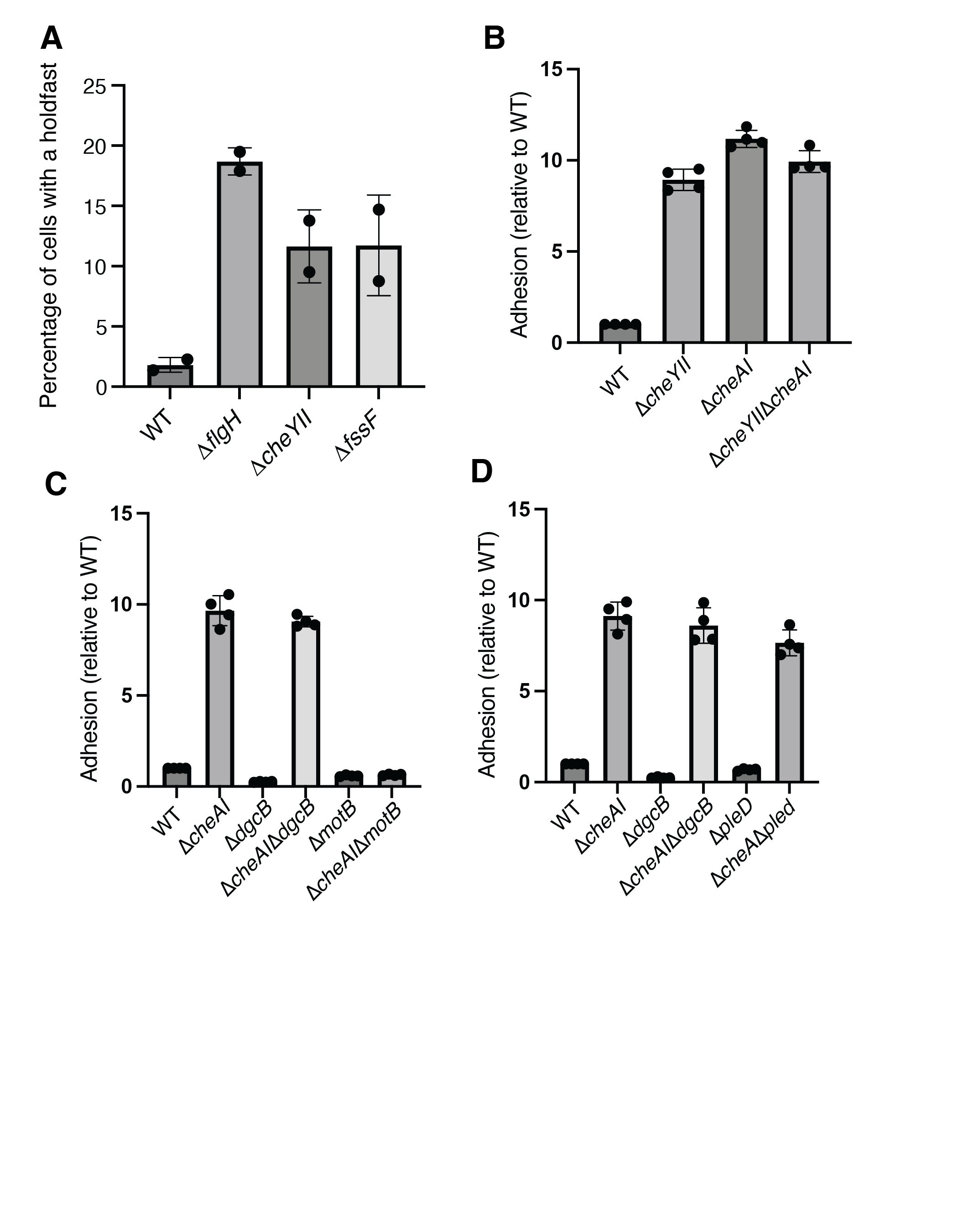


Figure S5: Chemotaxis mutants are hyperadhesive. (A) Quantification of holdfast staining microscopy. Percentage of cells associated with a holdfast depicted. (B), (C), and (D) CV staining of various ∆*cheAI* backgrounds to measure adhesion relative to wild-type.

Table S1: Strains used in this study

| Strain # | Organism | Phenotype | Description | Source |
| --- | --- | --- | --- | --- |
| DH103 | *C. crescentus* CB15 | WT | Wild type | ATCC 19089 |
| DH553 | *C. crescentus* CB15 | ∆*flgH* | In frame deletion of CC_2066 | Hershey *et al.* 2019 |
| DH649 | *C. crescentus* CB15 | ∆*pleD*∆*flgH* | In frame deletion of CC_2462 in DH553 background | Hershey *et al.* 2019 |
| DH659 | *C. crescentus* CB15 | ∆*pleD* | In frame deletion of CC_2462 | Hershey *et al.* 2019 |
| DH776 | *C. crescentus* CB15 | ∆*motB* | In frame deletion of CC_1573 | Hershey *et al.* 2019 |
| DH777 | *C. crescentus* CB15 | ∆*motB*∆*flgH* | In frame deletion of CC_1573 in DH553 background | Hershey *et al.* 2021 |
| DH787 | *C. crescentus* CB15 | ∆*dgcB* | In frame deletion of CC_1850 | Hershey *et al.* 2021 |
| DH788 | *C. crescentus* CB15 | ∆*flgH*∆*dgcB* | In frame deletion of CC_1850 in DH553 background | Hershey *et al.* 2021 |
| DH802 | *C. crescentus* CB15 | ∆*cheAI* | In-frame deletion of CC_0433 | This Work |
| DH814 | *C. crescentus* CB15 | ∆*cheYII* | In-frame deletion of CC_0437 | This Work |
| DH816 | *C. crescentus* CB15 | ∆*fliF* | In frame deletion of CC_0905 | Hershey *et al.* 2021 |
| DH827 | *C. crescentus* CB15 | ∆*cheAI* ∆*motB* | In frame deletion of CC_1573 in DH802 background | This Work |
| DH828 | *C. crescentus* CB15 | ∆*cheYII*∆*motB* | In frame deletion of CC_1573 in DH814 background | This Work |
| DH839 | *C. crescentus* CB15 | ∆*fliM* | In frame deletion of CC_2061 | This Work |
| DH847 | *C. crescentus* CB15 | ∆*cheYII*∆*pleD* | In frame deletion of CC_0437 in DH659 background | This Work |
| DH865 | *C. crescentus* CB15 | ∆*cheAI* ∆*pleD* | In-frame deletion of CC_0433 in DH659 background | This Work |
| DH869 | *C. crescentus* CB15 | ∆*cheAI*∆*dgcB* | In frame deletion of CC_1850 in DH802 background | This Work |
| DH1000 | *C. crescentus* CB15 | ∆*dgcB*∆*pleD* | In frame deletion of *CC_1850* integrated in DH659 background | This work |
| DH1124 | *C. crescentus* CB15 | ∆*fssF* | In frame deletion of CC_2175 | This Work |
| DH1125 | *C. crescentus* CB15 | ∆*fssF*∆*flgH* | In frame deletion of CC_2175 in FC1266 background | This Work |
| DH1129 | *C. crescentus* CB15 | ∆*fssF*∆*fliF* | In frame deletion of CC_2175 in DH816 background | This Work |
| DH1145 | *C. crescentus* CB15 | ∆*cheYII*∆*fssF* | In frame deletion of CC_2175 in DH814 background | This Work |
| DH1149 | *C. crescentus* CB15 | ∆*cheYII* ∆*cheAI* | In-frame deletion of *CC_0594* in DH802 background | This Work |
| DH1202 | *C. crescentus* CB15 | ∆*fssF* xyl::PfssF-empty | pDH1168 integrated at xylose locus of DH1124 | This Work |
| DH1203 | *C. crescentus* CB15 | ∆*fssF* xyl::PfssF-fssF | pDH1159 integrated at xylose locus of DH1124 | This Work |
| DH1350 | *C. crescentus* CB15 | *fssF*::*fssF*-venus | *venus* fused to 3' end of *fssf* at native *fssF* locus in DH103 background | This Work |
| DH1351 | *C. crescentus* CB15 | ∆*fliG* | In frame deletion of CC_0906 | This Work |
| DH1352 | *C. crescentus* CB15 | ∆*fliN* | in frame deletion of CC_0908 | This Work |
| DH1353 | *C. crescentus* CB15 | *fliM*::*fliM*-venus | *venus* fused to 3' end of *fliM* at native *fliM* locus in DH103 background | This Work |
| DH1354 | *C. crescentus* CB15 | *fssF*::*fssF*-venus Δ*fliG* | *venus* fused to 3' end of *fssF* at native *fssF* locusd in DH1351 background | This Work |
| DH1355 | *C. crescentus* CB15 | *fliG*::*fliG*-venus | *venus* fused to 3' end of *fliG* at native *fliG* locus in DH103 background | This Work |
| DH1356 | *C. crescentus* CB15 | *fssF*::*fssF*-venus Δ*fliN* | *venus* fused to 3' end of *fssF* at native *fssF* locus in DH1352 background | This Work |
| DH1357 | *C. crescentus* CB15 | *fliN*::*fliN*-venus | *venus* fused to 3' end of *fliN* at native *fliN* locusd in DH103 background | This Work |
| DH1358 | *C. crescentus* CB15 | *fssF*::*fssF*-venus ∆*fliM* | *venus* fused to 3' end of *fssf* at native *fssF* locus in DH839 background | This Work |
| DH1359 | *C. crescentus* CB15 | *fliN*::*fliN*-venus Δ*fliG* | *venus* fused to 3' end of *fliN* at native *fliN* locus in DH1351 background | This Work |
| DH1360 | *C. crescentus* CB15 | ∆*flgH* ∆*fssF* xyl::PfssF-fssF | pDH1168 integrated at xylose locus of DH1125 | This Work |
| DH1361 | *C. crescentus* CB15 | *fliM*::*fliM*-venus ∆*fliG* | *venus* fused to 3' end of *fliM* at native *fliM* locus in DH1351 background | This Work |
| DH1362 | *C. crescentus* CB15 | *fliG*::*fliG*-venus ∆*fliM* | *venus* fused to 3' end of *fliG* at native *fliG* locus in DH839 background | This Work |
| DH1363 | *C. crescentus* CB15 | *fliN*::*fliN*-venus ∆*fliM* | *venus* fused to 3' end of *fliN* at native *fliN* locus in DH839 background | This Work |
| DH1364 | *C. crescentus* CB15 | *fliG*::*fliG*-venus ∆*fssF* | *venus* fused to 3' end of *fliG* at native *fliG* locus in DH1124 background | This Work |
| DH1365 | *C. crescentus* CB15 | *fliN*::*fliN*-venus ∆*fssF* | *venus* fused to 3' end of *fliN* at native *fliN* locus in DH1124 background | This Work |
| DH1366 | *C. crescentus* CB15 | *fliM*::*fliM*-venus ∆*fliN* | *venus* fused to 3' end of *fliM* at native *fliM* locus in DH1352 background | This Work |
| DH1367 | *C. crescentus* CB15 | *fliM*::*fliM*-venus ∆*fssF* | In frame deletion of CC_2175 in DH1353 background | This Work |
| DH1368 | *C. crescentus* CB15 | ∆*flgH* ∆*fssF* xyl::PfssF-empty | pDH1159 integrated at xylose locus of DH1125 | This Work |
| DH1369 | *C. crescentus* CB15 | ∆*pleD*∆*flgH*∆*fssF* | In frame deletion of CC_2462 in DH1125 background | This Work |
| DH1370 | *C. crescentus* CB15 | ∆*flgH*∆*fssf*∆*motB* | In frame deletion of CC_1573 in DH1125 background | This Work |
| DH1371 | *C. crescentus* CB15 | ∆*pleD*∆*fssF* | In frame deletion of CC_2175 in DH659 background | This Work |
| DH1372 | *C. crescentus* CB15 | ∆*motB*∆*fssF* | In frame deletion of CC_2175 in DH776 background | This Work |
| DH1373 | *C. crescentus* CB15 | *fliG*::*fliG*-venus ∆*fliN* | *venus* fused to 3' end of *fliG* at native *fliG* locus in DH1352 background | This Work |
| DH1374 | *C. crescentus* CB15 | ∆*pleD*∆*dgcB*∆*fssF* | In frame deletion of CC_2175 in DH1000 background | This Work |
| DH1375 | *C. crescentus* CB15 | ∆*pleD*∆*motB*∆*fssF* | In frame deletion of CC_2175 in DH913 background | This Work |
| DH1376 | *C. crescentus* CB15 | ∆*cheYII*∆*dgcB* | In frame deletion of CC_1850 in DH1145 background | This Work |
| DH1377 | *C. crescentus* CB15 | ∆*flgH*∆*pleD*∆*dgcB* | In frame deletion of CC_1850 in DH649 background | This Work |
| DH1378 | *C. crescentus* CB15 | ∆*cheYII*∆*pleD*∆*dgcB* | In frame deletion of CC_1850 in DH847 background | This Work |
| DH1393 | *C. crescentus* CB15 | *fljK^T103C^* and *fssF*:*fssF-venus* | Insertion of *fssF-venus* into native *fssF* locus | This Work |

Table S2: Plasmids used in this study

| **Plasmid** | **Antibiotics** | **Publication description** | **Reference** |
| --- | --- | --- | --- |
| pDH418 | Km | pNPTS138-∆*flgH*; contains fusion of CC_2066 flanking regions with first and last 12 nucleotides of CC_2066 ORF included | Hershey *et al.* 2019 |
| pDH644 | Km | pNPTS138-∆*pleD*; contains fusion of CC_2462 flanking regions with first and last 12 nucleotides of CC_2462 ORF included | Hershey *et al.* 2019 |
| pDH762 | Km | pNPTS138-∆*cheAI*; contains fusion of CC_0433 flanking regions with first and last 12 nucleotides of CC_0433 ORF included | This work |
| pDH765 | Km | pNPTS138-∆*motB*; contains fusion of CC_1573 flanking regions with first and last 12 nucleotides of CC_1573 ORF included | Heshey *et al.* 2021 |
| pDH769 | Km | pNPTS138-∆*dgcB*; contains fusion of CC_1850 flanking regions with first and last 12 nucleotides of CC_1850 ORF included | Heshey *et al.* 2021 |
| pDH796 | Km | pNPTS138-∆*cheYI*I; contains fusion of CC_3472 flanking regions with first and last 12 nucleotides of CC_3472 ORF included | This work |
| pDH810 | Km | pNPTS138-∆*fliM*; contains fusion of CC_2008 flanking regions with first and last 12 nucleotides of CC_2008 ORF included | Heshey *et al.* 2021 |
| pDH1097 | Km | pNPTS138-fliM-venus; contains insertion of *venus* between the 3' end and downstream flanking region of CC_2008 | This work |
| pDH1114 | Km | pNPTS138-∆*fssF*; contains fusion of CC_2175 flanking regions with first 120 and last 12 nucleotides of CC_2175 ORF included | This work |
| pDH1159 | Km | pMT585 containing *PfssF* for integration at *xyl* locus; 101bp upstream of CC_2175 was inserted in reverse orientation into pMT585 | This work |
| pDH1168 | Km | pMT585 containing *fssF* under the control of *PfssF* for integration at *xyl* locus; 101bp upstream of CC_2175 fused to the CC_2175 ORF was inserted in reverse orientation into pMT585 | This work |
| pDH1379 | Km | pNPTS138-fliN-venus; contains insertion of *venus* between the 3' end and downstream flanking region of CC_0908 | This work |
| pDH1380 | Km | pNPTS138-fliG-venus; contains insertion of *venus* between the 3' end and downstream flanking region of CC_0906 | This work |
| pDH1381 | Km | pNPTS138-∆fliN; contains fusion of CC_0908 flanking regions with first and last 12 nucleotides of CC_0908 ORF included | This work |
| pDH1382 | Km | pNPTS138-∆fliG; contains fusion of CC_0906 flanking regions with first and last 12 nucleotides of CC_0906 ORF included | This work |
| pDH1383 | Km | pNPTS138-fssF-venus; contains insertion of *venus* between the 3' end and downstream flanking region of CC_2175 | This work |

Supplemental Table 1
